# Observation Interface of PDMS Membrane in a Microfluidic Chip Based on One-Step Molding

**DOI:** 10.3390/mi8030064

**Published:** 2017-02-23

**Authors:** Xiangyu Chen, Shuangyue Hou, Jian Chu, Ying Xiong, Penghui Xiong, Gang Liu, Yangchao Tian

**Affiliations:** 1 Department of Precision Machinery and Precision Instrumentation, University of Science and Technology of China, Hefei 230026, China; cxy0910@mail.ustc.edu.cn (X.C.); xph@mail.ustc.edu.cn (P.X.); 2 National Synchrotron Radiation Laboratory, Unitersity of Science and Technology of China, Hefei 230029, China; hsy58@mail.ustc.edu.cn (S.H.); xywch@ustc.edu.cn (Y.X.); 3 Institute of Materials, China Academy of Engineering Physics, Mianyang 621700, China; chujian@caep.cn

**Keywords:** microfluidic chip, polydimethylsiloxane (PDMS), PDMS membrane, imaging

## Abstract

Nowadays, researchers are focusing on sorting, characterizing and detecting micron or submicron particles or bacteria in microfluidic chips. However, some contradictions hinder the applications of conventional microfluidic chips, including the low working distance of high resolving power microscopy and the low light transmittance of conventional microfluidic chips. In this paper, a rapid and readily accessible microfluidic fabrication method is presented to realize observation with high magnification microscopy. With the one-step molding process, the interconnections, the thin observation interface of polydimethylsiloxane (PDMS) membrane and microfluidic channels were integrated into an intact PDMS replica. Three kinds of PDMS replicas with different auxiliary beams were designed and optimized by leakage experiments and analytical software. The observation interfaces of a 170 μm thickness PDMS membrane enlarges the application domain of microfluidic chips. By adopting a solution of high magnification observation, microfluidic devices could be applied widely in medical science, biology and material science.

## 1. Introduction

Microfluidic devices have an array of important applications in areas such as analytical systems [1,2], biomedical devices [3], material science [4], chemistry tools and biochemistry [5]. Methods of sorting, characterizing and detecting micron or submicron particles, cells and bacteria, particularly single cells in microfluidic chips, have been developed in recent years [6,7]. To obtain precise particle information and accurate cell signals in microfluidic channels, high-resolution microscopic observation and the detection of target particles were aimed towards integration within microfluidic chips. Since polydimethylsiloxane (PDMS) has great advantages including low costs, low auto-fluorescence and compatibility with many biological samples, it has become the most widely used material for building chip-based microfluidic devices [8,9]. Ordinarily, the thickness of these PDMS-based microfluidic devices is set to at least three millimeters [10,11] to ensure the interconnections work well with no leakage. However, the working distance of high magnification microscopy is generally less than 0.3 mm. The contradiction between the thickness of microfluidic chips and the working distance (W.D.) of high-resolution microscopy prevents PDMS microfluidic devices from being further exploited in many applications.

**Figure 1:**
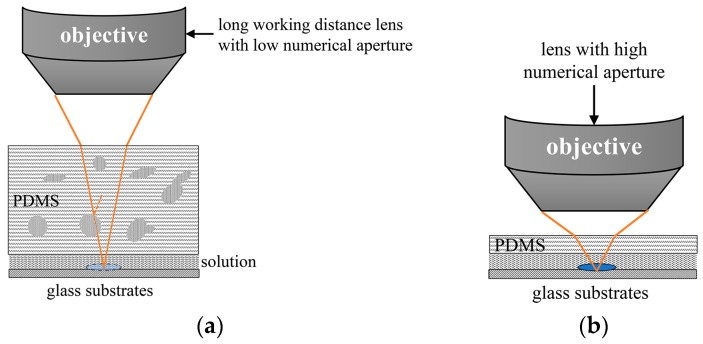
The scheme illustration of microscopic examinations: (**a**) observation of the particles in thick polydimethylsiloxane (PDMS)-based microfluidics; (**b**) observation of the particles in PDMS-based microfluidics with thin observation interface via the object of high numerical aperture.

With new development in microscopy techniques, some devices could be directly applied to the PDMS-based microfluidics. Although inverted microscopes are usually employed in biology labs [12], it was inappropriate to imagine the microfluidic chips with metal microelectrodes or non-transparent substrates [13]. While the long working distance objective has the ability to observe part of the thick PDMS microfluidic chips, the disadvantages have been universally acknowledged where they have: (1) a small visual field; (2) low numerical aperture (N.A.) resulting in low resolution according to the Rayleigh equation; (3) weak light collection; and (4) additional optical aberration. Therefore, it is currently not beneficial in detecting micron or submicron particles and bacteria, such as fluorescence detections and Raman measurements. Furthermore, as the PDMS thickness directly affects light transmission on the basis of the Beer-Lambert Law, the thicker the PDMS, the worse the transmittance becomes. PDMS is a two-part system of cross-linker/curing agent and siloxane [14]. Inadequate mixture, the natural attributes of the polymer and subsequent fabricating process (i.e., thermocompression) leads to nonuniformity of the thick PDMS block and leads to difficulties on focusing on microchannels in the thick PDMS microfluidic. The signals and information of submicron particles would not be captured in the microfluidic chips, particularly the weak signals of fluorescence or Raman (Figure 1a). In short, the thick PDMS block has become an issue that is hindering the application of microfluidic devices in biological and nanomaterial areas.

In addition to looking forward to the development of microscopy devices, microfluidic techniques are also essential in meeting the needs of the observation and detection of submicron particles. Perhaps thin and flat PDMS membranes have the potential for high-magnification observation and precise detection of submicron particles and bacteria in microfluidic channels. Jo et al. [15] reported a stacked multilayer 3-D micro-channel fabrication process. The top PDMS membrane was suitable for applications under high-magnification observation. However, fabricating glass or Si based microfluidic chips integrated with microelectrodes remains unavailable due to the compatibility of interconnections. Therefore, it is necessary to establish a rapid method to fabricate a thin and flat observation interface of the PDMS membrane to fulfill high-magnification observation and precise detection, which is also compatible with all kinds of microfluidic chips.

In the work described here, we present a one-step molding technique that allows the rapid and efficient construction of microfluidic chips with observation interfaces of thin PDMS membrane for high magnification light microscopy observation and detection. With the employment of tiny gaskets and glass cover plates, the PDMS-based microfluidic chip with observation interfaces less than 300 μm were constructed. In order to ensure reliable leak tightness, three kinds of PDMS replica structures with different auxiliary beams were designed, simulated and verified.

## 2. Materials and Methods

### 2.1. Design of Microfluidic Chips

**Figure 2:**
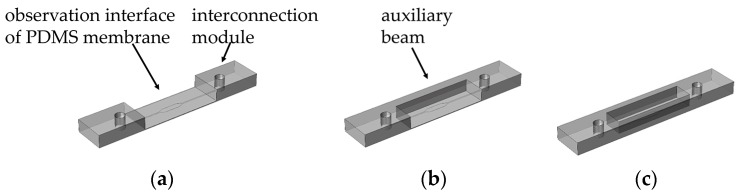
Three kinds of PDMS replicas structures with observation interface of PDMS membrane: (**a**) Replica A without auxiliary beam; (**b**) Replica B with one auxiliary beam; (**c**) Replica B with two auxiliary beams.

In biology labs, objects of high numerical aperture and flat commercial glass coverslips with a thickness of 170 μm are adopted in microscopic examination. To observe samples in the conventional thick microfluidic chips, objects of long working distance with low numerical aperture are employed to ensure sufficient working distance (Figure 1a). However, the small aperture angle leads to weak light collection. Moreover, the 3 mm non-uniform PDMS block hinders light transmission. The low numerical aperture and thick PDMS block both affect the observation of particles in microfluidics where the probable difficulty in focusing or image distortion would occur. To avoid these negative factors, the flat and thin observation interface of the PDMS membrane is expected to introduce the microfluidics chips (Figure 1b). In order to achieve the observation and detection of the particles in microfluidic channels with an object of high magnification and high numerical aperture, the thickness of the PDMS observation interface should be less than 300 μm. Meanwhile, the interconnections and microfluidic channels are expected to integrate into an intact PDMS replica for rapid fabrication with a single casting and demolding process. The design of an intact PDMS replica has a good compatibility with all kinds of substrates including non-flexibility substrates, which provides a wide range of applications. Figure 2a shows the ideal PDMS replica model for conveniently exchanging one objective for another. In consideration of the bonding process, the auxiliary beams are introduced to support the thin observation interface of PDMS membranes (Figure 2a,b).

### 2.2. Fabrication Process of One-Step Molding

**Figure 3:**
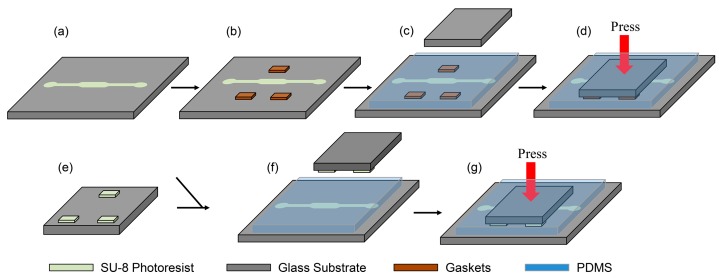
The scheme illustration of the two kinds of fabrication process of one-step molding: (**a**–**d**) the first process with silicon, polyvinyl chloride (PVC) and PDMS gaskets; (**e**,**f**) the second process with SU-8 gaskets. (**a**) microfluidic channel of SU-8; (**b**) gasket placement around the channel; (**c**) PDMS casting; (**d**) placement of the cover glass; (**e**) SU-8 gaskets; (**f**) PDMS casting; and (**g**) placement of the SU-8 glass wafer.

The one-step molding microfabrication technique for constructing PDMS replicas with an observation interface of the PDMS membrane was based on assembly molds (including pattern SU-8, several gaskets and a glass cover plate). Several materials of gasket with different thickness were tested including silicon, polyvinyl chloride (PVC), PDMS and SU-8. As shown in Figure 3, two kinds of fabrication techniques were adopted according to the materials of the gaskets. The first process was developed (Figure 3a–d), when silicon, PVC and PDMS were employed as separate gaskets. The representative channel mask for selective patterning was designed and printed. The central channel with a 2 mm width was the observation area and leakage test focus area. The negative-tone UV photoresist SU-8 2100 (MicroChem Corp., Westborough, MA, USA) was spin-coated on cleaned glass wafers of 65 mm diameter with a thickness of 105 μm (Figure 3a). Three smooth gaskets (2 mm × 4 mm) were placed around the observed channels area on the glass substrate (Figure 3b). The PDMS (Sylgard 184, Dow Corning, Midland, MI, USA) mixture, with a multiple adequate mix in the ratio of cross-linker/curing agent A:prepolymer B = 1:10, was degassed in a vacuum drying chamber. The PDMS mixture was poured into the horizontally placed molds. With the help of a portable vacuum suction pen, a 23 mm × 23 mm × 2 mm diameter glass cover plate with transparent scotch tape was gently placed into the PDMS mixture to prevent any bubbles forming at the interface (Figure 3c). The glass cover plate was adjusted to cover the three gaskets and object channel area and was firmly pressed into compact contact with the gaskets (Figure 3d). 

In the second process (Figure 3e–g), the SU-8 gaskets with thicknesses of 160 μm and 240 μm, respectively, were fabricated using photolithography techniques on glass wafers of 25 mm diameter (Figure 3e). Next, the glass wafer with the patterned SU-8 gaskets was placed into the PDMS mixture atop the channel area inversely (Figure 3f). The results were placed in an oven at 75 °C for 2 h to cure the PDMS. After the excess PDMS on the top surface of the glass cover plate, glass cover plate and gaskets was successfully removed, the PDMS replica was successfully released.

For studying the bonding process, PDMS replicas of 3 mm × 6 mm were cut to form three kinds of thin top observation structures. After placing in an acetone ultrasonic bath for 1 min, the PDMS replica and glass substrate were surface treated by oxygen plasma (30 W, 5 Pa, 30 s) in a reactive ion etching system. Immediately following the surface treatment, the PDMS replica was placed against the pretreated glass substrate where the bubbles were carefully and completely removed, before a conformal contact of the PDMS replica and glass substrate. A homemade clamp with flat plates (top, bottom) was adopted to fasten the PDMS replica and glass substrate and force to the central top aluminum plate was applied. Subsequently, the clamped results were placed in an oven at 90 °C for 1 h. The chips were deemed completed after cooling to ambient temperature.

Dielectrophoresis microfluidic chips were fabricated to compare and characterize the performance of observations. The glass substrate with a microelectrode layer and a microvoid array layer was fabricated using ultraviolet photolithography. The fabrication process of the glass substrate was similar to our previous work [16]. Subsequently, the PDMS replica was fabricated according to the one-step molding method. After bonding, copper wires were attached to the contact pads of the microelectrodes with a silver conductive paint (SPI No. 5002, SPI Supplies Inc., West Chester, PA, USA).

### 2.3. Leakage Test

The most common concern for microfluidic systems is concerns leakage. Therefore, microfluidic chips with three kinds of thin observation structures and conventional microfluidic chips without thin observations were tested for leakage. The silicone tubing (inner diameter 0.5 mm, outer diameter 2.1 mm) was directly plugged into the interconnections and red Rhodamine 6G (R6G) solution, which was employed for easy visualization, was introduced by peristaltic pump (BT100-2J, Baoding Longer Precision Pump, Ltd., Baoding, China) into the microfluidic channels for the leakage test. With an initial flow rate of 0.45 mL/h, the flow rates were systematically controlled and varied in increments of 0.45 mL/h at time intervals of 30 s until bond integrity was compromised. The flow rate at which the red solution started to leak was noted.

### 2.4. Experimental Procedure

The glass substrates with dielectrophoresis microelectrodes, the 3 mm PDMS dielectrophoresis microfluidics chip and the dielectrophoresis microfluidics chip with a 300 µm observation interface of PDMS membrane, were fabricated and set on the object stage of an upright fluorescence microscope (Nikon E600, Nikon, Tokyo, Japan) and upright perflectometer (Nikon Eclipse Ci-POL, Nikon) equipped with an objective of ×100/0.6 (W.D. 10 mm). A syringe pump (LSP01-1A, Baoding Longer Precision Pump, Ltd.) was used to pump test solutions into the dielectrophoresis microfluidic chips with observation interface. The images were captured by charge-coupled device (CCD) camera. A solution of 3 μm diameter polystyrene (PS) microspheres was injected into the microfluidic channel and the objective of ×100/0.6 was employed to observe the trapped results. A bacteria solution with a green fluorescence protein was also injected into another dielectrophoresis microfluidic chip with a 170 µm PDMS membrane. An objective of ×60/0.85 (W.D. 0.30 mm) was employed to observe and detect the fluorescence of the bacteria.

## 3. Results and Discussion

### 3.1. Optimization of Fabrication Process

**Figure 4:**
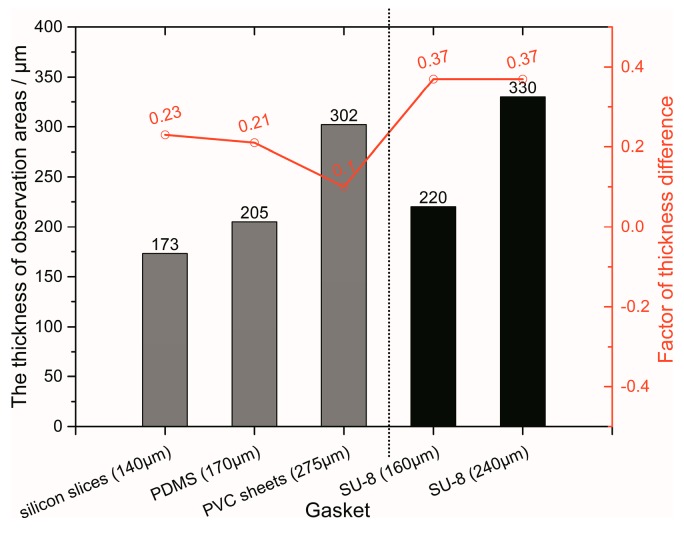
Gasket category and thickness with the corresponding thickness of the PDMS observation area.

The fabrication technique developed for microscopic observations was optimized through trial and error, and the improved method is put forward in this paper. Previously, releasing PDMS from the mold was difficult and time consuming. With the introduction of transparent scotch tape, the glass cover plate was removed with ease and the PDMS replica remained undamaged and perfect. In the fabrication process, silicon slices; PDMS slices; PVC sheets; and SU-8 slices were employed as gaskets. The materials and thicknesses of the gaskets and corresponding top PDMS observation areas are shown in Figure 4. The results of the two kinds of fabrication methods are also separately illustrated as grey and black columns. With less than 300 μm of PDMS membrane, PDMS replicas were compatible with most commercial high magnification lens. With silicon slice gaskets, a PDMS replica with the thinnest PDMS observation interfaces was successfully fabricated and included a 105 μm microchannel and an observation interfaces of only 168 μm, which is the same as a commercial glass coverslip. The thickness of the top PDMS observation area is closely related to the thickness of the gaskets. The thicker the gasket, the thicker the top PDMS observation area. The difference in thickness is actually the thickness of residual PDMS between the gaskets and glasses in the fabrication process. To evaluate the performance of the gaskets and fabrication methods, the factor of thickness difference (*f_t_*) was introduced to quantify the difference for each gasket and process. The equation of the factor of thickness difference is expressed as:

*f_t_ = (T_p_ − T_g_)/T_g_*, (1)

where *T_p_* and *T_g_* are the thickness of the PDMS observation areas and gasket, respectively. The factors of each gasket and method are also shown in Figure 4. Despite the top observation area being thickest with PVC gaskets, the thickness difference factor is lowest in the first fabrication technique. PVC exhibited the optimum performance as a gasket material due to the flatness and strong tolerance of the fabrication process; therefore, PVC was highly suitable for the first method due to its advantages, which include transparency, cleanness, low cost and ease of cutting and removal. In the second fabrication process, the thickness difference factors of the SU-8 gaskets were higher. The SU-8 gaskets were not ideally flat due to the spin-coat process on the small square glass wafers. Although the thicknesses of the SU-8 gaskets were different, the factors were both 0.37. This indicates that the thickness difference factor actually correlates with the gasket material and fabrication process. The factor was maintained within a small range, and when the confined material was adopted as the gaskets in the same fabrication method and experiment setting. With the aid of the factor, the second method appears to be significantly superior. By using a simple calculation, the SU-8 gaskets could provide customized PDMS replicas with top observation areas of the required thickness. Thus, PVC gaskets with an appropriate thickness are recommended for rapid batch manufacturing of PDMS replicas using the first method, with specialized fabrication by calculation provided for the second method.

With the use of gaskets and glass covers, an intact PDMS replica was obtained using the one-step molding process. This process is advantageous in saving time and improving yield. The construction of molds was interesting and anyone with any micro-fabrication experience can undertake the one-step molding process.

### 3.2. Selection of Auxiliary Beams

In order to select the stable replica, three kinds of PDMS replica structures with different auxiliary beams were tested, simulated and verified. The PDMS replicas with three kinds of thin observation interface were fabricated and leakage tests were conducted. The conventional microfluidic chip with the flat top surface was also tested as an experimental control, and no leakage occurred at a flow rate of 45 mL/h. The test solution oozed out of an inlet or the junction in Replica A, when the flow rate was 34 mL/h; whereas leakage occurred in a central channel in Replica B at a flow rate of 23 mL/h. No leakage occurred in Replica C when flow rate was as high as 45 mL/h. The results of the leakage test show that Replica C was performed the best out of the three kinds of PDMS replicas with thin observation structures. The flow rate of 45 mL/h in the leakage test experiments can be considered high throughput and enough to rinse the microfluidic chips. Therefore, Replica C is recommended as the PDMS structure for microfluidic chips. A key factor was the excellent contact between the PDMS replica and non-flexible substrate after the plasma process. For Replica A, the asymmetrical height of the PDMS top surface disrupted the formation of a good seal between the bottom surface and substrate. Furthermore, it was difficult to remove the junction of remnant air where it would further increase the deformation variable in the oven. While the structure of Replica B was beneficial in avoiding remnant air, it was inclined to bend with only one auxiliary beam. The observation area suffered from extrusion and the bond of the central channel failed. With the help of two auxiliary beams, Replica C was able to remain stable and a good seal, similar to the conventional flat PDMS replica, was formed.

**Figure 5:**
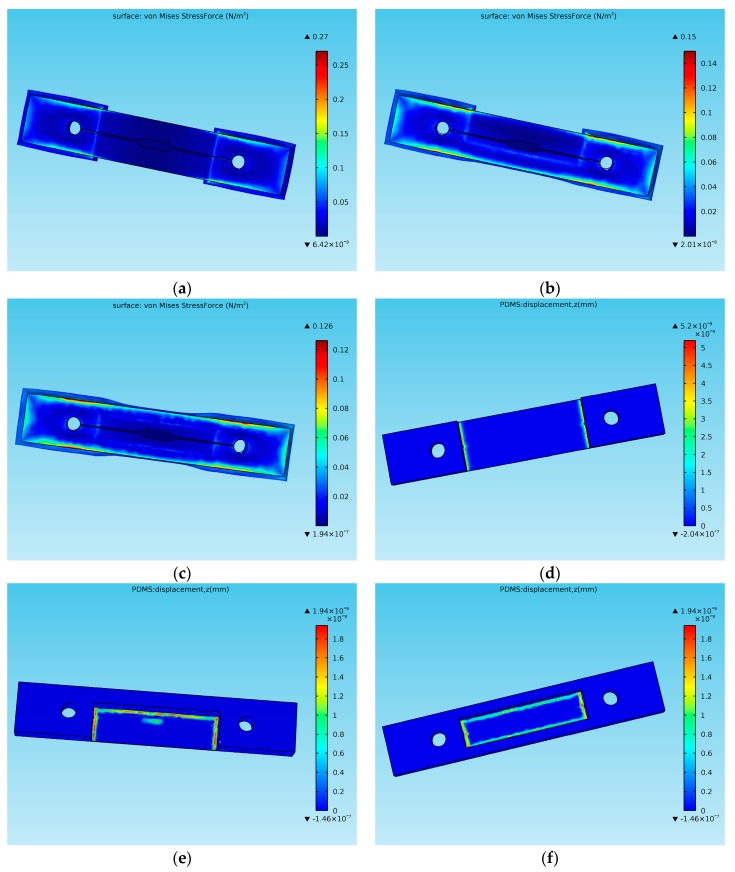
The simulation diagram of the stress and deformation in the *z*-direction of three kinds of PDMS replica structures in bonding process. Stress of bottom surface of: (**a**) Replica A; (**b**) Replica B; and (**c**) Replica C. Deformation in the *z*-direction of: (**d**) Replica A; (**e**) Replica B; and (**f**) Replica C.

To understand the effect of the homemade clamp in the bonding process, three kinds of PDMS replica structures were optimized by simulation in the finite element analysis method. A 3D Solid Mechanics interface was used to simulate the stress and deformation in the *z*-direction of the PDMS replicas in the clamps. The models, which contained a PDMS replica and glass substrate, were established. Three kinds of PDMS replicas were respectively placed on the glass substrates. The bottom surface of PDMS replicas contacted with glass substrates with no gaps. The Poisson ratio of the PDMS was 0.49 and the glass was 0.22. The Young’s modulus of PDMS was 750 kPa and the glass was 7.31 × 10^10^ Pa. In the *z*-direction, 1 × 10^−5^ N was applied to the upper surface of the PDMS. The simulation results of PDMS replicas are shown in Figure 5. For Replica A, the stress concentrated at the interconnection module, especially at the bottom (Figure 5a). With the introduction of the auxiliary beams, the stress dispersed to all replicas (Figure 5b,c). The deformation of the junction between the inlet and observation area was the maximum reached. As the values of the channel deformation on the junction are positive (Figure 5d–f), it means that it is possible for these junctions to divorce from the glass substrate or break down. The stress concentration and positive deformation values are important factors in influencing successful bonding and may explain the results of the leakage tests. Based on these results, Replica C is considered to be the optimal structure. Additionally, after the fabrication process, the auxiliary beams can be removed to conveniently exchange one objective for another.

### 3.3. Observation Results and Applications

**Figure 6:**
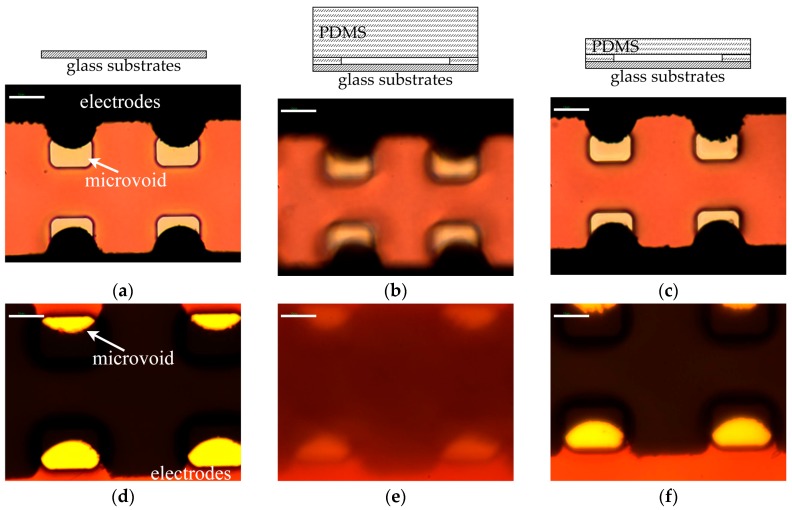
Photographs of Au microelectrodes with microvoids via the objective of ×100/0.6, scale bar: 10 µm. Microscope images with transmission illumination mode: (**a**) on the glass substrate; (**b**) on the microfluidics chip with a 3 mm thick PDMS block; and (**c**) on the microfluidics chip with a 300 µm observation interface of a PDMS membrane. Microscope images with reflection illumination mode: (**d**) on the glass substrate; (**e**) on the microfluidics chip with a 3 mm thick PDMS block; and (**f**) on the microfluidics chip with a 300 µm observation interface of the PDMS membrane.

In order to test and verify the performance of the observation interface of the PDMS membrane in the microfluidics, it was necessary to accomplish the observations of the same materials and structures with and without the PDMS membrane via the same objective and CCD. A batch of dielectrophoresis microfluidics chips with Au microelectrodes and microvoids were fabricated using the same mask and parameters. A glass substrate without PDMS; a 3 mm thick PDMS microfluidics chip with a microchannel of 100 µm height; and a 300 µm thick PDMS microfluidics chip with a microchannel of 100 µm height were respectively observed under a microscope with a transmission illumination mode and reflection illumination mode (Figure 6). In Figure 6a,d, the color and contrast are true and clear, and the brightness of the red photoresist (Figure 6a) and the Au electrodes (Figure 6d) was the highest. As shown in Figure 6b,e, image distortion occurred in the microfluidics with the 3 mm thick PDMS block. The edges of the electrodes and microvoids blurred and brightness drastically reduced. When the observation interface of the PDMS membrane with a 300 µm thickness was introduced, the images were still clear (Figure 6c,f); however, the brightness decreased by about 15% compared to the images of the glass substrate. Therefore, the thin and flat observation interface of the PDMS membrane has the ability to solve the issue of high magnification observation in microfluidic chips.

**Figure 7:**
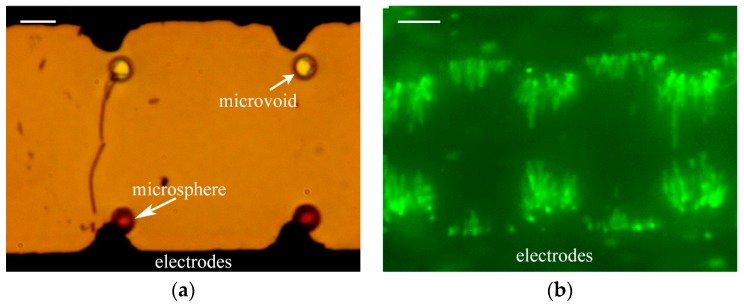
Photographs of two kinds of dielectrophoresis microfluidics chips with thin observation interfaces: (**a**) optical images of red trapped polystyrene (PS) microspheres in microfluidic chip, scale bar: 5 µm; and (**b**) fluorescence images of trapped bacteria in microfluidic chip, scale bar: 10 µm.

Thin observation interfaces were applied for the observation of two kinds of dielectrophoresis microfluidics chips. In Figure 7a, two red 3 μm diameter PS microspheres were respectively trapped in 3 μm microvoids below, which were in physical contact with the dielectrophoresis electrode. Figure 7b shows the fluorescence image, which was acquired by an objective of ×60/0.85. The bacteria were trapped on the edges of microelectrodes in the microfluidic channel. Individual bacteria were also clearly distinguished and the fluorescence intensity of individuals was slightly different. These show that microfluidic chips are reliable and the microscope images were relatively legible. It also indicates that the observation interfaces of PDMS membranes have the ability to achieve the observation and detection of micron or submicron particles and bacteria in microfluidic chips.

The integration of microfluidic chips with various substrates and channel geometries has been successfully fabricated. Furthermore, the observation interface of the PDMS membrane could be widely integrated in all kinds of PDMS-based microfluidic devices with suitable sizes. It is predicted that it is also compatible the most PDMS-based bonding processes. With strong irreversible bonding, the effective duration of microfluidic chips was longer than 60 days, and provides huge advantages in expanding applications for researchers without clean rooms.

## 4. Conclusions

In this study, the method of high magnification observation and detection of particles based on flat and observation interfaces of PDMS membrane less than 300 µm in microfluidic chips was presented. The one-step molding fabrication process was rapid, produced high yields and could be customized. Replica C was considered to be the optimal structure in the fabrication process. With the observation interface of the PDMS membrane, submicron particles and bacteria were successfully observed in microfluidic chips under ordinary microscopes equipped with objectives of high numerical aperture. Compatible with PDMS-based bonding processes and most substrates, this method could be widely applied in various sorts of microfluidic devices such as microfluidic electrophoresis chips and micro total analysis systems. A colorful and varied microcosmic world could be shown and may inspire more researchers to make use of the lab on a chip.
